# Understanding the barriers and facilitators to implementing and sustaining Mobile Overdose Response Services from the perspective of Canadian key interest groups: a qualitative study

**DOI:** 10.1186/s12954-024-00946-7

**Published:** 2024-02-02

**Authors:** Boogyung Seo, Nathan Rider, William Rioux, Adrian Teare, Stephanie Jones, Pamela Taplay, S. Monty Ghosh

**Affiliations:** 1 Department of Medicine, Faculty of Medicine and Dentistry, University of Alberta, Edmonton, AB Canada; 2 Cumming School of Medicine, University of Calgary, Calgary, AB Canada; 3 College of Medicine, University of Saskatchewan, Saskatoon, SK Canada; 4 Three Hive Consulting, Vancouver, BC Canada; 5 Grenfell Ministries, Hamilton, ON Canada; 6 Department of Internal Medicine, Faculty of Medicine and Dentistry, University of Alberta, Edmonton, AB Canada

**Keywords:** Fentanyl, Adulterants, Overdose crisis, Mobile Overdose Response Services, Supervised consumption

## Abstract

**Introduction:**

Unregulated supply of fentanyl and adulterants continues to drive the overdose crisis. Mobile Overdose Response Services (MORS) are novel technologies that offer virtual supervised consumption to minimize the risk of fatal overdose for those who are unable to access other forms of harm reduction. However, as newly implemented services, they are also faced with numerous limitations. The aim of this study was to examine the facilitators and barriers to the adoption of MORS in Canada.

**Methods:**

A total of 64 semi-structured interviews were conducted between November 2021 and April 2022. Participants consisted of people who use substances (PWUS), family members of PWUS, health care professionals, harm reduction workers, MORS operators, and members of the general public. Inductive thematic analysis was used to identify the major themes and subthemes.

**Results:**

Respondents revealed that MORS facilitated a safe, anonymous, and nonjudgmental environment for PWUS to seek harm reduction and other necessary support. It also created a new sense of purpose for operators to positively contribute to the community. Further advertising and promotional efforts were deemed important to increase its awareness. However, barriers to MORS implementation included concerns regarding privacy/confidentiality, uncertainty of funding, and compassion fatigue among the operators.

**Conclusion:**

Although MORS were generally viewed as a useful addition to the currently existing harm reduction services, it’s important to monitor and tackle these barriers by engaging the perspectives of key interest groups.

## Introduction

Proliferating supply of unregulated opioids (e.g., fentanyl) that is being contaminated with substances like xylazine and benzodiazepines continues to fuel the overdose crisis [1, 2]. The presence of these adulterants can exacerbate the severity of soft tissue injury, increase the risk of respiratory depression, and diminish the effects of naloxone [3]. Recently, there has been a rise in the concurrent use of opioids with methamphetamines, elevating the risk of a fatal overdose among people who use substances (PWUS) [4]. In 2023, Health Canada reported that over a half of apparent opioid toxicity deaths involved the use of a stimulant [5].

Numerous harm reduction and overdose response initiatives have emerged to mitigate the mortality and morbidity resulting from this crisis. Supervised Consumption Sites (SCS) are federally sanctioned facilities in Canada that provide safe and hygienic spaces for people to consume substances in the presence of a staff who is trained in overdose response [6]. 56(1) class exemption from *Controlled Drugs and Substances Act* has also authorized the provision of overdose prevention sites (OPS) [6], which are often low-barrier, temporary facilities that can be set up in trailers, vans, or pre-existing community organizations [6–8]. Take-home naloxone kits, safer supply, and needle exchange programs are also available as harm reduction methods to meet the diverse and complex needs of PWUS [9–11].

These strategies have been demonstrated to be effective at preventing fatal overdoses [6, 12], offsetting the costs related to emergency medical services [13, 14], and reducing the transmission of blood-borne diseases [15, 16]. In one study examining PWUS experience of OPS in Toronto, these facilities were seen as a “safe sanctuary” that fostered a sense of belonging and protection against violence and fear of arrest for PWUS [7]. Regardless, there are notable barriers that limit their wide-scale penetration and uptake. For instance, there is a prevailing belief that these services promote drug use and criminal activity [17, 18, 19]. They are often met with “Not in my backyard” type reactions, in which stigma against PWUS are transferred to the agencies that provide support and services to them [20]. Furthermore, while inhalation is one of the leading routes of opioid, cocaine, and methamphetamine consumption, many indoor SCS/OPS do not permit such practices, possibly failing to support a large demographic of PWUS [15].

In light of these shortcomings, novel technologies have been introduced to help fill some of the gaps in the currently existing harm reduction tools [21]. Mobile Overdose Response Services (MORS) are virtual technologies aimed at averting fatal drug overdoses, particularly for individuals who consume substances alone and/or lack immediate access to a physical facility for supervised consumption [22]. 

In this paper, MORS refer to a subset of overdose prevention services that come in the form of mobile applications or hotline services. Though spotting has historically been an informal practice of monitoring friends, acquaintances, or loved ones while they use substances, formal spotting services and organizations are now available [23, 24]. For example, National Overdose Response Service [25] and Brave [26] are hotline and mobile application services, respectively, in which clients can connect with an operator (ideally prior to consuming substances) who can initiate a personally tailored emergency response plan in the event of a suspected overdose [27, 28]. Both services operate across Canada in all provinces and territories with the former funded by Health Canada. Never Use Alone provides similar services in the United States, and currently operates without government funding. These services are often led by peer workers with lived experience of substance use, or those with personal connection to substance use disorder [27, 28]. Other formats, such as automated count-down systems are available, including Digital Overdose Response System (DORS) [29] and Connect by Lifeguard [30] app that will activate an emergency response unless the client manually resets the timer (only available in Alberta and British Columbia, respectively) [27, 28]. Another community-based harm reduction strategy is UnitedPhilly, a smartphone app based in Philadelphia that alarms nearby volunteers of an overdose who can then assist with naloxone administration [31].

To date, the authors are not aware of studies that have qualitatively explored the factors that serve as facilitators or barriers to implementing MORS. This study aimed to understand how best to develop MORS from the perspectives of Canadian key interest groups.

## Methods

### Participants

The interviewees consisted of PWUS, family members of PWUS, health care professionals, harm reduction workers, MORS operators, and the general public. PWUS were individuals who self-identified as using illicit and non-prescription substances (mainly opioids and methamphetamines). Health care professionals comprised of physicians, nurses, clinical educators, and managers of health care organizations. Harm reduction workers were employed in SCS, needle exchange program, or other harm reduction outreach services. MORS operators were from National Overdose Response Service hotline or the Brave app. Participants were recruited through a combination of snowball, purposive, and convenience sampling techniques. The first set of participants were known to the principal investigator and the MORS operations/management team, who were then requested to recommend another candidate in their existing network. The following inclusion criteria had to be met: (1) 18 years of age or older, (2) be able to communicate effectively in English, (3) identify with one of six key interest groups, and (4) able to provide informed consent.

### Interviews

The semi-structured interview guide was created in collaboration with MORS operators, individuals with lived experience of substance use, the research team, and various government and health officials. For each interview, verbal informed consent was obtained with the assurance that all information provided would remain confidential and anonymous. Supplementary mental health and addiction support were available in case participants felt distressed during the interview process; however, no participants accessed these supports.

A total of 64 interviews were conducted between November 2021 and April 2022 by evaluators from a third-party research organization specializing in qualitative research. The evaluators and participants were the only individuals present during the call and had no previously established relationship with one another. Prior to the interviews, participants were provided with a verbal overview regarding the various types of MORS to ensure a baseline understanding. Interviews ranged from 20 to 60 min and were all conducted over the telephone and recorded with *TapeACall*. An honorarium of $50 Canadian dollars was provided to PWUS only. 

### Coding and analysis

All interviews were completed prior to coding and analysis. Inductive thematic analysis informed by grounded theory [32, 33] was used to elucidate the major themes and subthemes that reflected the perceptions of participants regarding MORS. The third-party organization transcribed the data and performed the preliminary analysis of the results to minimize potential biases from the research team. Two evaluators (SJ and LA) with training in qualitative methods coded the transcripts using *Dedoose* software. They collectively inspected the first three transcripts to ensure alignment between the identified themes and afterwards, they independently coded half of the transcripts using a jointly created codebook. Throughout the process, each evaluator reviewed the transcripts coded by their counterpart to ensure the coding was congruent with the identified themes. Any discrepancies were resolved between the two evaluators and the principal investigator (MG). Once the initial coding was finished, the two evaluators reviewed a representative sample of coded quotations for each theme with a consulting project manager (KM). Member checking was conducted by sharing a paper format of the key themes with people with lived experience, MORS operators, and researchers to ensure that the data accurately reflected the perspectives of the key interest groups.

Results were reported using the Consolidated Criteria for Reporting Qualitative Research (COREQ) checklist. The study complies with the Tri-Council Policy Statement for Ethical Conduct for Research Involving Humans (TCPS 2) and the Helsinki Declaration [34]. It was approved by the University of Calgary Conjoint Health Research Ethics Board (REB21-1655).

## Results

A total of 64 interviewees consisted of twenty-five PWUS, five family members of PWUS, ten health care professionals, six harm reduction workers, six MORS operators, and twelve members of the general public. The provinces and territories in which the participants resided can be found in Table 1. The following demographic information only pertains to PWUS. The mean age was 38.52±12.02, ranging from 20 to 66 years of age. Ten individuals self-identified as women, fourteen as men, and one as non-binary. Three resided in a rural area. Of eight themes elucidated from our interviews, four pertained to each of potential barriers and facilitators. The following themes were identified: (1) privacy and confidentiality, (2) funding and service capacity, (3) burnout and compassion fatigue among operators, (4) access to technology, (5) stigma and nonjudgmental environment, (6) anonymity, (7) marketing strategies, and (8) discovering a new sense of purpose.

**Table 1:** Area of residence for each key interest group

	PWUS (*n* = 25)	Family member of PWUS (*n* = 5)	Health care professionals (*n* = 10)	Harm reduction workers (*n* = 6)	MORS operators (*n* = 6)	General public (*n* = 12)
AB	8	4	8	3	2	7
BC	0	0	1	0	0	3
SK	0	0	0	1	0	0
ON	15	0	0	0	2	1
QC	1	1	0	0	0	0
Atlantic	1	0	1	1	1	0
Territories	0	0	0	1	1	1
Urban	22	5	8	4	6	10
Rural	3	0	2	2	0	2

### Barriers to MORS implementation

***Theme 1*** Concerns regarding data privacy and confidentiality

There was a prevalent concern among interviewees regarding the stringency of privacy measures and the security of personal details that were identified as barriers to using MORS. One participant worried as to “*whether somebody is going to tap into their phone*” (MORS Operator 06, AB) and another questioned if the apps “*might be hijacked*” (General Public 01, AB). A few participants proposed that these services need to be free from government oversight to properly allay concerns regarding the protection of their personal information.

“Some people are still nervous to use virtual services because they believe it's all being backlogged somewhere that they can't see you know?” (General Public 06, BC).

“Well, I think once again, for Alberta, there’s a lack of trust in how information is used by anything that has been implemented through the Alberta government.” (Family Member 03, AB).

Moreover, it was deemed imperative that clients knew “*exactly what information is being shared, and who it’s being shared with*” (Health care Professional 08, AB). Respondents highlighted how any confidentiality agreements should disclose what type of information is shared with the police or emergency medical services (EMS). Some were also not in favor of MORS requiring access to a health care number to become a client (which is required as per the policies at SCS in Alberta), which some individuals do not have access to.

“Being really clear about your confidentiality agreements. And also very clear boundaries on what you do disclose to like police, of EMS. Because like there's certain things you have to disclose, but a lot of the time like, it seems kind of vague. And just making sure the people using the virtual services know exactly what information is being shared, and like who it's being shared with.” (Health care Professional 08, AB)

“Also, if you’re accessing the virtual consumption site, what information do you need to provide? Because if it – like I know that the government is in the process of making it so that you have to use an Alberta health care number, like a lot of our patients don’t have that either so that’s a huge barrier.” (Health care Professional 06, AB)

“If they’re afraid that their information is going to be, you know, if it’s going to be on their permanent then people can see that they’re accessing those services they’re going to not use that. And I think that’s in particular for people who are, you know, returning to drug use or have legal involvement, you know. They don’t want to be traceable so I think that that’s an issue.” (Health care Professional 01, AB)

***Theme 2*** Service capacity and uncertainty of future funding

It was brought to attention that MORS are increasingly being accessed by PWUS for mental health and addiction support. Operators expressed concerns regarding the capacity to attend to each client and the implications on staffing if the calls extended beyond 20–30 min, which is possible with mental health-related calls. Although it is positive news that MORS are gaining traction for services beyond harm reduction, participants theorized that this could pose problems in regard to costs and resourcing.

“A call that we do is about 20 min each – 25 to – maybe 20 to 30. And if we expand anymore within our mandate, these calls can go into hours. And then that’s not effective, because we only have so many people on the line.” (MORS Operator 02, ON)

“Because I feel like NORS (National Overdose Response Service) is really going to pick up steam, and eventually, at some point, I'm sure the calls will be outweighing the people that are able to answer at that time.” (MORS Operator 03, AB)

Although some participants shared the benefits of providing mental health support through MORS, a few interviewees emphasized that invasive check-ins for mental health could get *“too in people’s grills and [drive] them away”* (Harm Reduction Worker 06, AB). Some clients may appreciate the connection they have with PWUS who have experience of substance use, but it was felt that appropriate professional boundaries still needed to be respected.

***Theme 3*** Lack of proper and consistent access to technology

It was also revealed that one of the biggest key factors limiting adoption of MORS is the access to proper technology. Especially in rural areas, respondents often struggled with inconsistent cell and internet service. As operators anticipated a greater demand for MORS with increased advertising efforts, some recommended more government support to provide better technological access.

“I think, like as I said before, like it's great for people who have a phone and like have access to Wi-Fi and have like a safe place to use. Because like even if like our street-based population has a phone they're probably going to be using in an alley or like somewhere that like they don't know the address. And it's like not really a whole lot beneficial.” (Health care Professional 08, AB)

“It comes back to the phone. We can’t reach people who don’t have a phone, so. When you’re on the street, it’s really hard to keep a phone on you. And if you’re in a rural community there is crappy reception.” (MORS Operator 02, ON)

“Yeah, but on the rural side I think it would mainly be productivity, and reception, just maybe an uptake in using technology, right? People that maybe live out in a rural area, just the thought of virtual just doesn’t even make sense to them. Technology literacy, I guess.” (General Public 07, AB)

***Theme 4 ***Addressing burnout and compassion fatigue among MORS operators

While the operators themselves did not identify any negative impacts of working at MORS, some respondents pointed out potential pitfalls. The most notable risks were trauma and stress associated with responding to overdose. Some respondents warned against how repeated exposure to these situations may ultimately lead to burnout and compassion fatigue, which could negatively impact workforce retention and the sustainability of MORS.

“I can just imagine how stressful, and how like angsty it is. And you're on the other line watching someone overdose. And all you can do is wait until paramedics get there. That causes a lot of trauma.” (PWUS 17, NS)

Respondents shared currently existing strategies aimed at supporting the operators, including debriefing with supervisors in the case of a suspected overdose. Frequent check-ins with operators were deemed essential to “*make sure that they’re feeling* […] *that they don’t feel triggered*” (MORS Operator 06, AB). The support team typically consisted of people with lived experience, clinicians, and counselors to guide operators through challenging situations. Operators noted how these steps helped them feel supported when volunteering or working for MORS.

“They’re also open to, you know, shoot me a message, shoot me an email, give me a call if you have a concern. If there has ever been a dramatic situation where, let’s say, somebody has experienced on the call somebody who’s had an overdose and we’ve had to call the ambulance, they're always super-quick to say, “Message me, let’s debrief. Give me a phone call. Do you need to talk? Like, let’s work through this.” The support has always been there, right from the very beginning, so it’s been really awesome.” (MORS Operator 03, AB)

Despite the frequent check-ins, respondents expressed how there could be improved connections between the daily operations and the management team to ensure better communication and collaboration around policy development.

“Ensure that upper management is always around to be supportive […] so sometimes the upper, upper management is disconnected from what we do on a day-to-day level. And then sometimes policies are set and then they have to be reset, because it doesn’t – it’s not – it definitely – it’s not in the line of what we do – of what we practice daily.” (MORS Operator 02, ON)

In terms of training, most respondents who have worked or volunteered for MORS stated they received adequate training related to (1) overdose response, (2) psychosis de-escalation, and (3) mental health first aid and suicide prevention. On the contrary, a few participants felt that the harm reduction education of staff was particularly important and perhaps somewhat overlooked.

“Well, I would think the training we’re doing now is working pretty well. The truth is, like, I don’t know, the situations are pretty unique, and so is each person that navigates them. So, at present there’s a one-size shoe that fits all for training. Like, we offer de-escalation training and things like that, but yes.” (MORS Operator 01, ON)

“And being just resource-savvy, I think is the other key. It’s – you know, a lot of times it’s not about knowing exactly what the clients need, but knowing what resources are potentially available to help get the client to where they need to be, is important.” (MORS Operator 06, AB)

### Facilitators to MORS implementation

***Theme 5*** Facilitating a stigma and judgment-free environment

In general, PWUS who had previous experience with hotline-based MORS reported a positive attitude toward the service, particularly in regard to talking to another peer operator in an inclusive, trauma-informed environment. As MORS are often operated and led by people with lived experience of substance use, it was believed that this could be leveraged to increase its adoption.

“I’ve had a bit of a rough time not too long ago and I was on my own at the time and just kind of feeling a little bit low, just wanted somebody to chat with basically. And I found the experience really well, the people on the line were very compassionate, they’re, there wasn't a kind of judgmental tone, which was nice. And I wasn't even sure what I was getting into at the time, so I thought it was, the experiences that I've had have been nice, have been comforting and not, and not feeling, made me feel any worse or anything like that or these types of situations, that's for sure.” (PWUS 13, ON)

“Yes. Definitely. Honestly, the no judgement thing is probably a big thing. They were amazing. I don’t think I had a single person who judged me – there was nothing said or done, anything – single person on the line – nobody, I don’t think.” (PWUS 11, ON)

***Theme 6 ***Ensuring anonymity and dignity of PWUS during substance use

Although concerns surrounding privacy breach was previously listed as a possible barrier to MORS utilization, some respondents believed that these virtual services can ensure better anonymity while consuming substances. Current MORS operators put forth suggestions on how to strengthen these measures, such as (1) recruiting operators from various regions across Canada (to reduce the possibility of connecting clients to operators from their social circles), (2) using mobile apps or hotlines to limit face-to-face interaction, (3) implementing a code system using the two letters of first and last name and birth year, (4) use of pseudonyms or nicknames, (5) not calling back clients without their explicit consent, and (6) ensuring a proper destruction of any paperwork containing personal information.

Given these additional measures, respondents revealed that they would be less concerned about ensuring their anonymity when using virtual services compared to physical sites. They emphasized how these services allowed them to consume substances without being exposed or scrutinized in public spaces as they “*didn’t want embarrassment [and] just didn’t want people to know*” (PWUS 10, ON). In addition, MORS were deemed beneficial for small, rural communities where people may require additional measures to ensure their anonymity due to stigma and alienation associated with substance use.

“I think it’s the anonymity […] rather than asking in person, there’s something to be said for being able just to talk on the phone and no one see your face or know your name if you're inquiring about these types of supports.” (Harm Reduction Worker 05, AB)

"Privacy is a big one because like I said many people aren’t comfortable using in a group or with other people, and many people they’re keeping these things like a secret for their family as well, right, which is understandable. So privacy for sure, health and safety knowing that someone’s looking out for you” (General Public 03, ON)

***Theme 7 ***Increasing public awareness of MORS through advertising and promotion

While operators believed that MORS currently have the capacity to accommodate most clients, they felt that more funding and support would be needed if more clients used the services. One operator expressed how long-term, reliable funding for MORS was contingent on the political climate (MORS Operator 01, ON).

“I think just again, you know, community – people are going to see that and get their backs up. They're not going to like the idea of it, again because they feel like it’s promoting people using substances, when again, they just don’t understand. I would say that was going to be the biggest issue.” (MORS Operator 03, AB)

Moreover, respondents discussed how greater financial support would allow MORS to develop a better marketing campaign to promote these services throughout the country. As relatively new interventions, MORS were seen as needing more financial assistance going forward to increase its awareness and to inform the public of its roles, in efforts to facilitate a more comfortable conversation around substance use in general and helping to minimize the stigma and shame.

“I'm trying to make this a comfortable conversation so that other people that are struggling don't have to feel that they're being shamed and that they can't talk about it you know? This should be a conversation that we're having as a society.” (Family Member 02, AB)

Displaying posters in community clinic offices, housing facilities, public washrooms, television, and social media platforms (e.g., Facebook, Instagram, and YouTube) were recommended as strategies for improving awareness of MORS among the laypeople. Additionally, being endorsed by governing bodies and public institutions such as Health Canada, police services, provincial health authorities, and EMS were seen as acceptable ways to improve its penetration on a wider scale.

“So you know like you go to a restaurant and you know in the bathroom how they have the posters on the back of the bathroom doors? It needs to be there. It needs to be at the door to the bar. It needs to be everywhere.” (Family Member 02, AB)

“I would like to see more promotion within – I'm in Alberta, so within Alberta Health Services, in their addiction and mental health offices. I run into quite a few counsellors that don’t actually know anything about DORS, which is kind of unbelievable to me. But [we need] to see more messaging servicing this. I don’t see a lot of it in rural areas” (Harm Reduction Worker 05, AB)

“The more we promote to people that we care about them, that we accept that you are using drugs for whatever reason you use them for […] and as a health care professional I want you to know that I want you to be safe and I will support whatever decision you make and I’ll try to keep you as safe as I can. So I feel like this virtual supervised consumption site will – or services will help that aspect” (Health care Professional 06, AB)

***Theme 8 ***Creating a sense of purpose and promoting professional development

MORS operators indicated how working and volunteering at harm reduction organizations have added profound insight to their understanding of substance use and their ability to help others in need. One respondent emphasized how their work created a sense of purpose *“during some of the darkest times in the pandemic”* (MORS Operator 01, ON). MORS also provided an opportunity for operators to acquire new sets of skills that enabled them to continue working in the harm reduction field and social work.

“When I stumbled upon this information to volunteer, it was just like a breath of fresh air for me. I felt like I could sit in my home, and I could help somebody from, you know, the other side of the country and, you know, be able to be there and support them. So, I've been able to help others while still feeling like I’m doing something for myself, because this is my passion. I truly enjoy working with individuals who use substances and have mental health concerns, that’s my alley. So, it’s been almost a saving grace for me of, I'm still holding on to that, while I'm not employed, kind of thing. So, it’s been fantastic.” (MORS Operator 03, AB).

Lastly, participants highlighted the sense of community and belonging while working at MORS. This included providing peer support to operators if they were encountering personal issues. For instance, one operator recalled an experience where another colleague helped them achieve sobriety. MORS organizations were generally regarded as safe spaces that accepted and cared for its operators.

“Because they make you a part of their family, if you're having trouble with anything, they will help you out no matter what. I lost one of my jobs and they pretty much were like, we'll figure it out and I've never felt so secure with a bunch of drug addicts in my life.” (MORS Operator 04, NS)

## Discussion

This study is the first to qualitatively examine the factors that may influence the adoption and implementation of MORS in Canada. Participants discussed one-on-one peer support and anonymity as external systems-based facilitators. External systems-based barriers included data privacy/confidentiality, funding/resource availability, and access to proper technologies. Internal program-based facilitators included discovering a sense of purpose and making a positive contribution to the harm reduction community while reducing barriers such as occupational burnout.

Consistent with the findings in the current literature [27], interviewees noted possible breach of privacy and confidentiality as a factor that would discourage PWUS from using MORS. Moreover, PWUS expressed hesitancy especially toward government-sanctioned harm reduction services.

This is a well-warranted concern, given that legal concerns surrounding child custody, housing, and employment among PWUS have been cited in the literature as barriers to seeking help or calling EMS during a medical emergency [35, 36]. For instance, housing policies in many jurisdictions across Canada allow landlords to evict residents for substance use, possibly pushing these vulnerable populations into homelessness [37]. On the contrary, some PWUS showed preference for MORS over physical facilities in order to safeguard their anonymity since virtual services do not require video or face-to-face communication. Still, respondents stressed the need for improved transparency in how and where the client information will be used, stored, and managed. To ensure a better protection of personal identity, respondents suggested using pseudonyms, communication platforms requiring only audio, and secure destruction of any paperwork that contains sensitive client information. The authors believe that this can significantly increase the uptake of MORS among PWUS, particularly if these measures are adequately communicated.

Today, the types of services offered at SCS and OPS have significantly diversified beyond supervised consumption and many clients are now able to access new drug paraphernalia, wound care, mental health support, and referrals to other social services [38]. This phenomenon of service expansion and diversification are similarly observed with MORS [39], demonstrating the wide-ranging and complex needs among PWUS. According to a recent study published by Rider et al. (2023), MORS has adopted novel purposes such as methamphetamine de-escalation, education on safer substance use, mental health support, and more [39]. This is in line with the experiences shared by the operators in this study, who are catering to the individual needs of each client through one-on-one support. Additionally, being able to connect with an operator with shared experience of substance use was a significant motivator for PWUS to use MORS. Indeed, PWUS from previous studies described their interactions with peer workers as less stigmatizing, nonjudgmental, and conducive to trust-building compared to non-peer workers [40, 41]. As a result of this established trust, PWUS also reported an enhanced sense of safety while using substances in the presence of peer workers [41].

While it is optimistic news that more PWUS are inclined to seek help through these services, the widening scope of MORS will require careful monitoring of volunteer and staff availability, as well as the potential onboarding of more operators who will be available to address the unique needs of each client.

The fate of many harm reduction services depends on the political landscape of local jurisdictions [42], as noted by some participants. The funding and maintenance for harm reduction programs are uncertain as substance use has been historically perceived as a criminal behavior rather than a health issue [43]. In 2021, the Alberta government announced that they would be closing the only SCS located in Calgary, which has been “highly disruptive to the neighborhood” and relocate its services to more appropriate, pre-existing facilities [44]. It is important to note that public health policies are often shaped by the public’s sentiment toward the issue [45], but even a brief exposure to evidence-based information has been shown to modestly increase support for harm reduction services among the general public [46]. As such, the provision of more educational resources and marketing of non-stigmatizing messages regarding MORS and substance use in general may need to be considered.

Contrary to our findings, recent studies have reported increased ownership of mobile devices among the unhoused population [47]. For instance, cellphone ownership among PWUS in downtown Eastside of Vancouver was estimated to be 48%, with a majority indicating interest in utilizing mobile applications for overdose response [48]. Despite this, practical problems such as connection to Wi-Fi/cell service, theft, and limited battery life could still persist as sources of limitation, especially for those who are unhoused [38]. Furthermore, those who reside in rural/remote areas may still struggle to access these services due to unreliable cell service [49]. While young unhoused individuals are more likely to use telehealth and automated phone interventions, older individuals are less likely to do so due to lower digital literacy and psychological aging [38]. MORS should investigate ways to reach people who lack access to mobile devices or struggle to use conventional applications.

A survey of peer responders in British Columbia revealed a high compassion satisfaction and low burnout even during the Covid-19 pandemic, which was attributed to reasonable workload, fair pay, and receiving appropriate recognition for their performance [50]. This finding aligns with the sentiment expressed by the operators in our study, in which employment at MORS was seen as an opportunity to positively contribute to the community and discover a new sense of purpose. Although there are many perceived benefits of working for MORS, there remains pitfalls to be addressd. Staff turn-over is often a hindrance to achieving fidelity and penetration of many social services [51]. Especially when the concept of “task shifting” has been widely adopted to mitigate the staffing shortages in various health care domains [52], the risk of burnout and compassion fatigue remains a significant risk among those working on the front lines of the overdose crisis [35, 53].

It is crucial that appropriate strategies are implemented in such a high-stress work environment to mitigate these limitations. Based on a study that examined work conditions in United States substance use treatment centers, job autonomy, support for creativity, and performance-based rewards were key factors that have improved counselor retention [54]. Operator debriefing and engagement are also key to the improvement of hotline-specific MORS going forward, and have been cited as important in many other health care settings [55]. Future implementation of MORS should prioritize engaging the perspectives of operators to ensure workplace conditions adequately support their mental health and safety.

### Strengths, limitations, and future directions

One strength of this study is the large sample size of participants recruited from various regions of Canada. However, the use of snowball and convenience sampling methods may have excluded many viable and relevant participants. Nonetheless, this study did collate the viewpoints of those most familiar with MORS, and we believe that such individuals are best suited to understand the nuances involved in MORS implementation. It is important to recognize that the barriers and facilitators to adopting harm reduction services, including MORS, may be context-dependent and vary between urban and rural areas. Future studies should strive to elucidate the unique challenges that serve as deterrents to implementing MORS specifically in rural and under-resourced communities of Canada. There still remains a need to quantitatively examine the service satisfaction and utility of MORS from client and operator perspectives.

## Conclusion

The complex and ever-changing nature of substance use disorder necessitates novel technologies and methods to address its concerns. While MORS may be a helpful and much-needed adjunct service to bridge the gaps in the standard harm reduction tools, its scope and limitations must be continuously explored to ensure its sustainability.

## Data Availability

The data that support the findings of this study are available on request from the corresponding author, M.G. The data are not publicly available due to the sensitivity of substance use and interview transcripts containing information that could compromise the privacy of research participants.

## References

[1] Karamouzian M, Rafat B, Kolla G, Urbanoski K, Atkinson K, Bardwell G (2023). Challenges of implementing safer supply programs in Canada during the COVID-19 pandemic: a qualitative analysis. Int J Drug Policy.

[2] Russell C, Law J, Bonn M, Rehm J, Ali F (2023). The increase in benzodiazepine-laced drugs and related risks in Canada: the urgent need for effective and sustainable solutions. Int J Drug Policy.

[3] Boon M, Van Dorp E, Broens S, Overdyk F (2020). Combining opioids and benzodiazepines: effects on mortality and severe adverse respiratory events. Ann Palliat Med.

[4] Palis H, Xavier C, Dobrer S, Desai R, Sedgemore K, Scow M (2022). Concurrent use of opioids and stimulants and risk of fatal overdose: a cohort study. BMC Public Health.

[5] Health Canada. Opioid- and Stimulant-related Harms—Canada.ca [Internet]. 2023 [cited 2023 Jul 19]. https://health-infobase.canada.ca/substance-related-harms/opioids-stimulants/

[6] Health Canada. Supervised consumption explained: types of sites and services [Internet]. 2018 [cited 2023 Jul 31]. https://www.canada.ca/en/health-canada/services/substance-use/supervised-consumption-sites/explained.html

[7] Foreman-Mackey A, Bayoumi AM, Miskovic M, Kolla G, Strike C (2019). ‘It’s our safe sanctuary’: experiences of using an unsanctioned overdose prevention site in Toronto, Ontario. Int J Drug Policy.

[8] Blythe S, Chapman L, Dodd Z, Gagnon Z, Hobbs H, Westfall J. This tent saves lives: how to open an overdose prevention site;2017.

[9] Choremis B, Campbell T, Tadrous M, Martins D, Antoniou T, Gomes T (2019). The uptake of the pharmacy-dispensed naloxone kit program in Ontario: a population-based study. PLoS ONE.

[10] Milaney K, Haines-Saah R, Farkas B, Egunsola O, Mastikhina L, Brown S (2022). A scoping review of opioid harm reduction interventions for equity-deserving populations. Lancet Reg Health Am.

[11] Haines M, O’Byrne P (2023). Safer opioid supply: qualitative program evaluation. Harm Reduct J.

[12] Suen LW, Wenger LD, Morris T, Majano V, Davidson PJ, Browne EN (2023). Evaluating oxygen monitoring and administration during overdose responses at a sanctioned overdose prevention site in San Francisco, California: a mixed-methods study. Int J Drug Policy.

[13] Khair S, Eastwood CA, Lu M, Jackson J (2022). Supervised consumption site enables cost savings by avoiding emergency services: a cost analysis study. Harm Reduct J.

[14] Chambers LC, Hallowell BD, Zang X, Rind DM, Guzauskas GF, Hansen RN (2022). The estimated costs and benefits of a hypothetical supervised consumption site in Providence, Rhode Island. Int J Drug Policy.

[15] Ali F, Russell C, Kaura A, Leslie P, Bayoumi AM, Hopkins S (2023). Client experiences using a new supervised consumption service in Sudbury, Ontario: a qualitative study. PLoS ONE.

[16] Health Canada. aem. 2020 [cited 2023 Jul 28]. Canadian supervised consumption sites statistics—Canada.ca. https://health-infobase.canada.ca/datalab/supervised-consumption-sites-blog.html

[17] Parkes T, Price T, Foster R, Trayner KMA, Sumnall HR, Livingston W (2022). ‘Why would we not want to keep everybody safe?’ The views of family members of people who use drugs on the implementation of drug consumption rooms in Scotland. Harm Reduct J.

[18] Strike C, Watson TM, Kolla G, Penn R, Bayoumi AM (2015). Ambivalence about supervised injection facilities among community stakeholders. Harm Reduct J.

[19] Atkinson AM, McAuley A, Trayner KMA, Sumnall HR (2019). ‘We are still obsessed by this idea of abstinence’: A critical analysis of UK news media representations of proposals to introduce drug consumption rooms in Glasgow, UK. Int J Drug Policy..

[20] Kolla G, Strike C, Watson TM, Jairam J, Fischer B, Bayoumi AM (2017). Risk creating and risk reducing: community perceptions of supervised consumption facilities for illicit drug use. Health Risk Soc.

[21] Loverock A, Marshall T, Viste D, Safi F, Rioux W, Sedaghat N, Kennedy M, Ghosh SM (2023). Electronic harm reduction interventions for drug overdose monitoring and prevention: A scoping review. Drug Alcohol Depend..

[22] Rioux W, Marshall T, Ghosh SM (2023). Virtual overdose monitoring services and overdose prevention technologies: opportunities, limitations, and future directions. Int J Drug Policy.

[23] Perri M, Guta A, Kaminski N, Bonn M, Kolla G, Bayoumi A (2023). Spotting as a risk mitigation method: a qualitative study comparing organization-based and informal methods. Int J Drug Policy.

[24] Perri M, Kaminski N, Bonn M, Kolla G, Guta A, Bayoumi AM (2021). A qualitative study on overdose response in the era of COVID-19 and beyond: how to spot someone so they never have to use alone. Harm Reduct J.

[25] NORS. NORS. [cited 2024 Jan 10]. National Overdose Response Service. https://www.nors.ca/about

[26] Brave. Brave Coop [Internet]. [cited 2024 Jan 17]. https://brave.coop/

[27] Marshall T, Viste D, Jones S, Kim J, Lee A, Jafri F (2023). Beliefs, attitudes and experiences of virtual overdose monitoring services from the perspectives of people who use substances in Canada: a qualitative study. Harm Reduct J.

[28] Matskiv G, Marshall T, Krieg O, Viste D, Ghosh SM (2022). Virtual overdose monitoring services: a novel adjunctive harm reduction approach for addressing the overdose crisis. Can Med Assoc J.

[29] DORS [Internet]. [cited 2024 Jan 17]. Digital overdose response system. https://www.dorsapp.ca

[30] Lifeguard Digital Health Inc. | Lifeguard Digital Health [Internet]. [cited 2024 Jan 17]. https://lifeguarddh.com/

[31] Schwartz DG, Ataiants J, Roth A, Marcu G, Yahav I, Cocchiaro B (2020). Layperson reversal of opioid overdose supported by smartphone alert: a prospective observational cohort study. EClinicalMedicine.

[32] Braun V, Clarke V (2006). Using thematic analysis in psychology. Qual Res Psychol.

[33] Glaser BG, Strauss AL. Discovery of grounded theory: strategies for qualitative research. 2017.

[34] World Medical Association (2013). World medical association declaration of Helsinki: ethical principles for medical research involving human subjects. JAMA..

[35] Follett KM, Piscitelli A, Parkinson M, Munger F. Barriers to calling 9–1–1 during overdose emergencies in a Canadian context. Crit Soc Work. 2019;15(1)

[36] Moallef S, Choi J, Milloy MJ, DeBeck K, Kerr T, Hayashi K (2021). A drug-related Good Samaritan Law and calling emergency medical services for drug overdoses in a Canadian setting. Harm Reduct J.

[37] Fleming T, Collins AB, Boyd J, Knight KR, McNeil R (2023). “It’s no foundation, there’s no stabilization, you’re just scattered”: a qualitative study of the institutional circuit of recently-evicted people who use drugs. Soc Sci Med.

[38] DeLaCruz-Jiron EJ, Hahn LM, Donahue AL, Shore JH (2023). Telemental health for the homeless population: lessons learned when leveraging care. Curr Psychiatry Rep.

[39] Rider N, Safi F, Marshall T, Jones S, Seo B, Viste D (2023). Investigating uses of peer-operated Virtual Overdose Monitoring Services (VOMS) beyond overdose response: a qualitative study. Am J Drug Alcohol Abuse.

[40] Kennedy MC, Boyd J, Mayer S, Collins A, Kerr T, McNeil R (2019). Peer worker involvement in low-threshold supervised consumption facilities in the context of an overdose epidemic in Vancouver. Canada Soc Sci Med.

[41] Bardwell G, Kerr T, Boyd J, McNeil R (2018). Characterizing peer roles in an overdose crisis: preferences for peer workers in overdose response programs in emergency shelters. Drug Alcohol Depend.

[42] Foreman-Mackey A, Pauly B, Ivsins A, Urbanoski K, Mansoor M, Bardwell G (2022). Moving towards a continuum of safer supply options for people who use drugs: a qualitative study exploring national perspectives on safer supply among professional stakeholders in Canada. Subst Abuse Treat Prev Policy.

[43] Kulesza M, Teachman BA, Werntz AJ, Gasser ML, Lindgren KP (2015). Correlates of public support toward federal funding for harm reduction strategies. Subst Abuse Treat Prev Policy.

[44] Pearson H, Gilligan M. Global News. 2021 [cited 2023 Sep 10]. Calgary’s supervised consumption site at Sheldon Chumir centre to close, services being relocated—Calgary | Globalnews.ca. https://globalnews.ca/news/7900839/calgary-sheldon-chumir-supervised-consumption-site-relocation/

[45] Burstein P (2003). The impact of public opinion on public policy: a review and an agenda. Polit Res Q.

[46] Strickland JC, Victor G, Ray B (2022). Perception of resource allocations to address the opioid epidemic. J Addict Med.

[47] Heaslip V, Richer S, Simkhada B, Dogan H, Green S (2021). Use of technology to promote health and wellbeing of people who are homeless: a systematic review. Int J Environ Res Public Health.

[48] Tsang VWL, Papamihali K, Crabtree A, Buxton JA (2021). Acceptability of technological solutions for overdose monitoring: perspectives of people who use drugs. Subst Abuse.

[49] RahimipourAnaraki N, Mukhopadhyay M, Wilson M, Karaivanov Y, Asghari S (2022). Virtual healthcare in rural and remote settings: a qualitative study of Canadian rural family physicians’ experiences during the COVID-19 pandemic. Int J Environ Res Public Health.

[50] Mamdani Z, McKenzie S, Ackermann E, Voyer R, Cameron F, Scott T (2023). The cost of caring: compassion fatigue among peer overdose response workers in British Columbia. Subst Use Misuse.

[51] Woltmann EM, Whitley R (2007). The role of staffing stability in the implementation of integrated dual disorders treatment: an exploratory study. J Ment Health.

[52] Olding M, Boyd J, Kerr T, McNeil R (2021). “Anda we just have to keep going”: Task shifting and the production of burnout among overdose response workers with lived experience. Soc Sci Med..

[53] Shearer D, Fleming T, Fowler A, Boyd J, McNeil R (2019). Naloxone distribution, trauma, and supporting community-based overdose responders. Int J Drug Policy.

[54] Knudsen HK, Johnson JA, Roman PM (2003). Retaining counseling staff at substance abuse treatment centers: effects of management practices. J Subst Abuse Treat.

[55] Azizoddin DR, Vella Gray K, Dundin A, Szyld D (2020). Bolstering clinician resilience through an interprofessional, web-based nightly debriefing program for emergency departments during the COVID-19 pandemic. J Interprof Care.

